# Supplementary material for: New Statistical Methods for Constructing Robust Differential Correlation Networks to characterize the interactions among microRNAs
Source: Sci Rep. 2019 Mar 5;9:3499. doi: 10.1038/s41598-019-40167-8 (PMC6401044; doi:10.1038/s41598-019-40167-8)
Supplement: Supplementary file 1 — Supplementary_Document I and Supplementary_Document II [file 41598_2019_40167_MOESM1_ESM.pdf]

New Statistical Methods for Constructing Robust Differential Correlation Networks to characterize the interactions among microRNAs

Supplementary Documents I

Danyang Yu[1], Zeyu Zhang[2], Kimberly Glass[3], Jessica Su[3], Dawn L. DeMeo[3], Kelan Tantisira[3], Scott T. Weiss[3], Weiliang Qiu[3*]

[1] Department of Information and Computing Science, College of Mathematics and Econometrics, Hunan University, Hunan, China

[2] Bioinformatics Department, School of Life Sciences and Technology, Tongji University, Shanghai, China

[3] Channing Division of Network Medicine, Brigham and Women's Hospital/ Harvard Medical School, Boston, USA

[*] Corresponding author's email address: stwxq@channing.harvard.edu

1. Proof of $\sum_{i=1}^{n_0+n_1}(y_i - \bar{y})\, w_i \propto \widehat{\rho_1} - \widehat{\rho_2}$

We have

$$w_i = \begin{cases} \dfrac{n_1(x_i - \overline{x_1})(z_i - \overline{z_1})}{\sqrt{\sum_{y_j=1}(x_j - \overline{x_1})^2 \sum_{y_j=1}\left(z_j - \overline{z_1}\right)^2}} & y_i = 1, \\ \dfrac{n_0(x_i - \overline{x_0})(z_i - \overline{z_0})}{\sqrt{\sum_{y_j=0}(x_j - \overline{x_0})^2 \sum_{y_j=0}\left(z_j - \overline{z_0}\right)^2}} & y_i = 0, \end{cases}$$

n_1 represents the number of cases, n_0 represents the number of controls, $y_i = 1$ indicates the *i*-th subject is a case, $y_i = 0$ indicates the *i*-th subject is a control,

$$\sum_{i=1}^{n_0+n_1} (y_i - \bar{y})\, w_i = \sum_{y_i=1} \left(y_i - \frac{n_1}{n_0+n_1}\right) w_i + \sum_{y_i=0} \left(y_i - \frac{n_1}{n_0+n_1}\right) w_i$$
$$= \sum_{y_i=1} w_i - \frac{n_1}{n_0+n_1} \sum_{y_i=1} w_i - \frac{n_1}{n_0+n_1} \sum_{y_i=0} w_i$$
$$= \frac{n_0}{n_0+n_1} \sum_{y_i=1} w_i - \frac{n_1}{n_0+n_1} \sum_{y_i=0} w_i$$
$$= \frac{n_0 n_1}{n_0+n_1} (\widehat{\rho_1} - \widehat{\rho_2})$$

2.The formulas for ST3

$$\mathrm{U}^{\mathrm{III}} = \sum_{i=1}^{n_0+n_1} (y_i - \bar{y})\, \mathrm{w}_i^{\mathrm{III}}$$

In the following, we will derive the formula for $\mathrm{w}_i^{\mathrm{III}}$.

Denote

$M_{x_1} = \, median\ of\ x_i$ where $y_i = 1,\ M_{z_1} = \, median\ of\ z_i$ where $y_i = 1,$

$M_{x_0} = \, median\ of\ x_i$ where $y_i = 0,\ M_{z_0} = \, median\ of\ z_i$ where $y_i = 0.$

Denote the within-group absolute deviations as

$$Q_i^{x_1} = |x_i - M_{x_1}| \;\; where\ y_i = 1, Q_i^{z_1} = |z_i - M_{z_1}| \;\; where\ y_i = 1,$$

$$Q_i^{x_0} = |x_i - M_{x_0}| \;\; where\ y_i = 0, Q_i^{z_1} = |z_i - M_{z_0}| \;\; where\ y_i = 0.$$

Denote the ordered within-group absolute deviations as

$$Q_{(1)}^{x_1} \le \cdots \le Q_{(n_1)}^{x_1}, Q_{(1)}^{z_1} \le \cdots \le Q_{(n_1)}^{z_1}, Q_{(1)}^{x_0} \le \cdots \le Q_{(n_0)}^{x_0}, Q_{(1)}^{z_0} \le \cdots \le Q_{(n_0)}^{z_0}$$

Denote the $100(1-\beta)$ percentiles of the within-group absolute deviations as

$$\widehat{\omega}_{x_1} = Q_{(m_1)}^{x_1} \;, \widehat{\omega}_{z_1} = Q_{(m_1)}^{z_1} \;, \widehat{\omega}_{x_0} = Q_{(m_0)}^{x_0} \;, \widehat{\omega}_{z_0} = Q_{(m_0)}^{z_0},$$

where $m_1 = (1-\beta)n_1, m_0 = (1-\beta)n_0,\ and\ \beta = 0.2.$

Next, we find the quantiles of the within-group standardized random variables:

$$i_1^{x_1} \text{ is the number of } x_i \text{ values such that } \frac{(x_i - M_{x_1})}{\widehat{\omega}_{x_1}} < -1 \;\; y_i = 1$$

$$i_1^{z_1} \text{ is the number of } z_i \text{ values such that } \frac{(z_i - M_{z_1})}{\widehat{\omega}_{z_1}} < -1 \; y_i = 1$$

$$i_1^{x_0} \text{ is the number of } x_i \text{ values such that } \frac{(x_i - M_{x_0})}{\widehat{\omega}_{x_0}} < -1 \;\; y_i = 0$$

$$i_1^{z_0} \text{ is the number of } z_i \text{ values such that } \frac{(z_i - M_{x_0})}{\widehat{\omega}_{z_0}} < -1 \;\; y_i = 0$$

$$i_2^{x_1} \text{ is the number of } x_i \text{ values such that } \frac{(x_i - M_{x_1})}{\widehat{\omega}_{x_1}} > 1 \;\; y_i = 1$$

$$i_2^{z_1} \text{ is the number of } z_i \text{ values such that } \frac{(z_i - M_{z_1})}{\widehat{\omega}_{z_1}} > 1 \; y_i = 1$$

$$i_2^{x_0} \text{ is the number of } x_i \text{ values such that } \frac{(x_i - M_{x_0})}{\widehat{\omega}_{x_0}} > 1 \;\; y_i = 0$$

$$i_2^{z_0} \text{ is the number of } z_i \text{ values such that } \frac{(z_i - M_{x_0})}{\widehat{\omega}_{z_0}} > 1 \;\; y_i = 0$$

Then, we calculate trimmed within-group sums:

$$S_{x_1} = \sum_{i=i_1^{x_1}+1}^{n_1 - i_2^{x_1}} x_{(i)} \quad y_i = 1, S_{z_1} = \sum_{i=i_1^{z_1}+1}^{n_1 - i_2^{z_1}} z_{(i)} \quad y_i = 1,$$

$$S_{x_0} = \sum_{i=i_1^{x_0}+1}^{n_0 - i_2^{x_0}} x_{(i)} \quad y_i = 0, S_{z_0} = \sum_{i=i_1^{z_0}+1}^{n_0 - i_2^{z_0}} z_{(i)} \quad y_i = 0$$

We next calculate adjusted trimmed within-group means:

$$\hat{\phi}_{x_1} = \frac{\widehat{\omega}_{x_1}\left(i_2^{x_1} - i_1^{x_1}\right) + S_{x_1}}{n_1 - i_2^{x_1} - i_1^{x_1}}, \qquad \hat{\phi}_{z_1} = \frac{\widehat{\omega}_{z_1}\left(i_2^{z_1} - i_1^{z_1}\right) + S_{z_1}}{n_1 - i_2^{z_1} - i_1^{z_1}},$$

$$\hat{\phi}_{x_0} = \frac{\widehat{\omega}_{x_0}\left(i_2^{x_0} - i_1^{x_0}\right) + S_{x_0}}{n_0 - i_2^{x_0} - i_1^{x_0}}, \qquad \hat{\phi}_{z_0} = \frac{\widehat{\omega}_{z_0}\left(i_2^{z_0} - i_1^{z_0}\right) + S_{z_0}}{n_0 - i_2^{z_0} - i_1^{z_0}}$$

We then calculate within-group scaled random variables:

$$U_i = \begin{cases} \dfrac{x_i - \hat{\phi}_{x_1}}{\widehat{\omega}_{x_1}} & y_i = 1 \\ \dfrac{x_i - \hat{\phi}_{x_0}}{\widehat{\omega}_{x_0}} & y_i = 0 \end{cases}, \qquad V_i = \begin{cases} \dfrac{z_i - \hat{\phi}_{z_1}}{\widehat{\omega}_{x_1}} & y_i = 1 \\ \dfrac{z_i - \hat{\phi}_{z_0}}{\widehat{\omega}_{z_0}} & y_i = 0 \end{cases}$$

We next make sure the within-group scaled random variables are within the range [-1, 1]:

$$A_i = \varphi(U_i), B_i = \varphi(V_i),$$

where $\varphi(\mathrm{x}) = \max[-1, \min(1, \mathrm{x})]$.

Finally, we define $\mathrm{w}_i^{\mathrm{III}}$ as the product of group size and the sample correlations based on the within-group scaled random variables:

$$\mathrm{w}_i^{\mathrm{III}} = \begin{cases} \dfrac{n_1 A_i B_i}{\sqrt{\sum_{y_j=1}^{n_1} A_j^2 \sum_{y_j=1}^{n_1} B_j^2}} & y_i = 1 \\ \dfrac{n_0 A_i B_i}{\sqrt{\sum_{y_j=1}^{n_0} A_j^2 \sum_{y_j=1}^{n_0} B_j^2}} & y_i = 0 \end{cases}$$

We can obtain

$$\mathrm{var}(\mathrm{U}^{\mathrm{III}}) = \bar{y}(1-\bar{y}) \sum_{i=1}^{n_0+n_1} (w_i^{III} - \bar{w}^{\mathrm{III}})^2$$

$$\bar{w}^{\mathrm{III}} = \sum_{i=1}^{n_0+n_1} \frac{\mathrm{w}_i^{\mathrm{III}}}{n_0 + n_1}$$

$$T^{\mathrm{III}} = \frac{\mathrm{U}^{\mathrm{III}}}{\mathrm{var}(\mathrm{U}^{\mathrm{III}})} \xrightarrow{H_0^{III}} \chi_1^2.$$

3.The definition of a g-and-h-distribution

Let *Z* be a random variable having the standard normal distribution. Then the random variable W(Z; g, h) constructed below follows a g-and-h distribution:

$$W(Z; g, h) = \begin{cases} \frac{\exp(gZ)-1}{g} \exp\left(\frac{hZ^2}{2}\right) & g > 0 \\ Z\exp\left(\frac{hZ^2}{2}\right) & g = 0 \end{cases}.$$

4. The pre-processing of the real miRNA data GSE15008

There are 1,614 probes in the GSE15008 dataset, including miRNAs, control probes, and negative controls (blank). We first drew (1) the boxplots of the expression levels of all hsa-miRNAs, (2) the boxplots of the expression values of miRNAs with "SPOT_ID" equal to "control:50%DSMO", and (3) the boxplots of the expression values of miRNAs with "SPOT_ID" equal to "BLANK" (Figure S1). We then calculated the median expression level for each of the 3 groups of miRNAs. For miRNAs with duplicated observations, we kept the one having largest average expression value. After this cleaning, 538 hsa-miRNAs kept. Finally, we kept 178 hsa-miRNAs with expression values of all subjects larger than the median expression level of control probes.

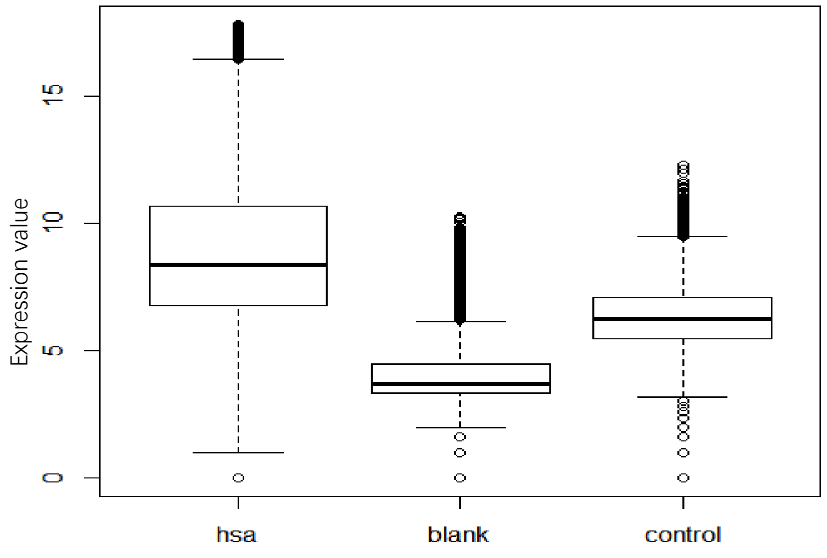

Figure-S1 boxplots of expression values of 3 groups of probes in the GSE15008 dataset.

5.The quantile plots of GSE15008 after data cleaning

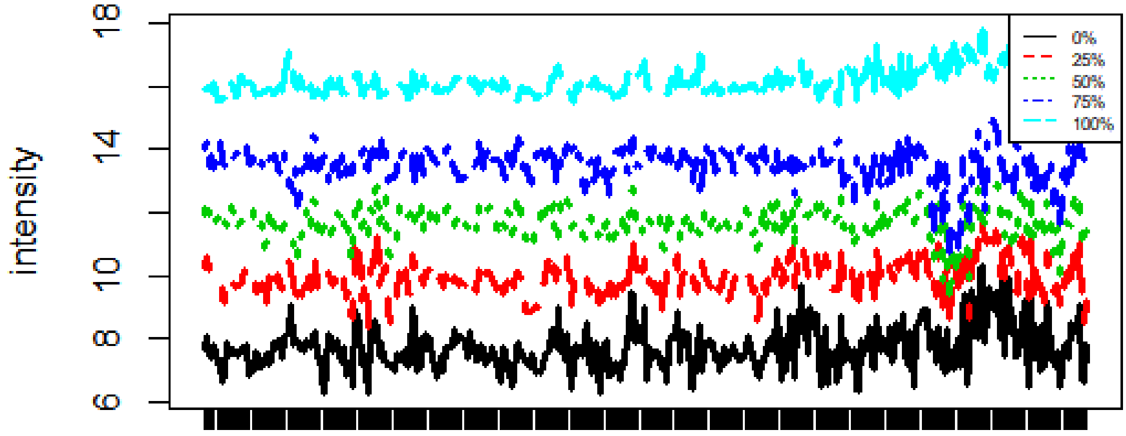

Figure-S2 Plot of percentiles of the miRNA expression levels across samples after data cleaning for the GSE15008 dataset

6.The scatter plot of the first principal component (PC1) versus the second principal component (PC2) of GSE15008 after data cleaning

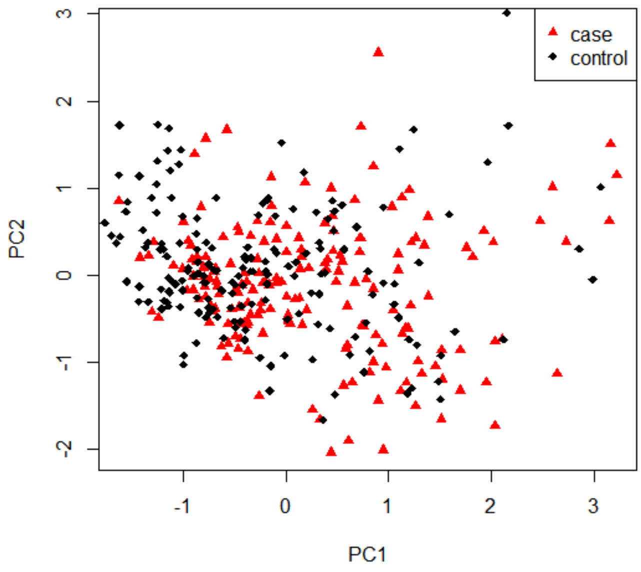

Figure-S3 Scatter plot of the first principal component (PC1) versus the second principal component (PC2).

7. The parallel boxplots of tests in all scenarios in simulation studies

Please see the compressed file figure_S4.zip

8. Table of ranks of powers in all scenarios and the scenarios including twopcor and twocor

Please see the file table_S1.xlsx

9. Table of targeted genes of hubs detected in real analysis obtained by miRSystem

Please see the file table_S2.xlsx

10. Table of Functional Annotation of hubs detected in real analysis obtained by miRSystem

Please see the file table_S3.xlsx

New Statistical Methods for Constructing Robust Differential Correlation Networks to characterize the interactions among microRNAs

Supplementary Document II

Danyang Yu[1], Zeyu Zhang[2], Kimberly Glass[3], Jessica Su[3], Dawn L. DeMeo[3], Kelan Tantisira[3], Scott T. Weiss[3], Weiliang Qiu[3*]

[1] Department of Information and Computing Science, College of Mathematics and Econometrics, Hunan University, Hunan, China

[2] Bioinformatics Department, School of Life Sciences and Technology, Tongji University, Shanghai, China

[3] Channing Division of Network Medicine, Brigham and Women's Hospital/ Harvard Medical School, Boston, USA

[*] Corresponding author's email address: stwxq@channing.harvard.edu

A Deriviation of the asymptotic distribution of the ST6 test statistic

Let Y_i denote the disease status of subject i, where $i = 1, 2, ..., n$, $n = n_0 + n_1$, n_0 is the number of the non-diseased subjects (controls, $Y_i = 0$) and n_1 is the number of the diseased subjects (cases, $Y_i = 1$).

Let's consider the following logistic regression model

$$logit\left[Pr\left(Y_i = 1 \left| w_i^{III}, w_i^{IV}\right.\right)\right] = \gamma_0 + \gamma_1 w_i^{III} + \gamma_2 w_i^{IV}. \tag{A1}$$

We would like to test the composite hypotheses $H_0^{VI} : \gamma_1 = \gamma_2 = 0$ versus $H_a : \gamma_1 \neq 0$ or $\gamma_2 \neq 0$.

The log-likelihood function of the logistic regression (A1) is

$$l(\boldsymbol{\Theta}) = \sum_{i=1}^{n} y_i \left(\gamma_0 + \gamma_1 w_i^{III} + \gamma_2 w_i^{IV}\right) - \log\left[1 + \exp\left(\gamma_0 + \gamma_1 w_i^{III} + \gamma_2 w_i^{IV}\right)\right],$$

where $\boldsymbol{\Theta} = (\gamma_0, \gamma_1, \gamma_2)^T$.

The score statistics are partial derivatives of the log-likelihood function with respect ot the parameters of interest, evaluated at the values postulated by the null hypothesis $H_0^{VI} : \gamma_1 = \gamma_2 = 0$.

We have

$$\begin{aligned} \frac{\partial l(\boldsymbol{\Theta})}{\partial \gamma_0} &= \sum_{i=1}^{n} (y_i - \pi_i), \\ \frac{\partial l(\boldsymbol{\Theta})}{\partial \gamma_1} &= \sum_{i=1}^{n} w_i^{III} (y_i - \pi_i), \\ \frac{\partial l(\boldsymbol{\Theta})}{\partial \gamma_2} &= \sum_{i=1}^{n} w_i^{IV} (y_i - \pi_i), \end{aligned}$$

where

$$\pi_i = Pr(Y_i = 1 | w_i^{III}, w_i^{III}) = \frac{\exp(\gamma_0 + \gamma_1 w_i^{III} + \gamma_2 w_i^{IV})}{1 + \exp(\gamma_0 + \gamma_1 w_i^{III} + \gamma_2 w_i^{IV})}.$$

Under $H_0^{VI} : \gamma_1 = \gamma_2 = 0$,

$$\pi_i \overset{H_0^{VI}}{=} \frac{\exp(\gamma_0)}{1 + \exp(\gamma_0)} \equiv \pi_0.$$

Let $\partial l(\boldsymbol{\Theta}) / \partial \gamma_0 = 0$ under H_0^{VI}. We got an estimate of π_0:

$$\hat{\pi}_0 = \bar{y} = \sum_{i=1}^{n} y_i / n.$$

Hence, the score statistics are

$$U^{III} = \frac{\partial l(\mathbf{\Theta})}{\partial \gamma_1}\bigg|_{\pi_0=\bar{y},\gamma_1=\gamma_2=0} = \sum_{i=1}^{n} w_i^{III}(y_i - \bar{y}),$$
$$U^{IV} = \frac{\partial l(\mathbf{\Theta})}{\partial \gamma_2}\bigg|_{\pi_0=\bar{y},\gamma_1=\gamma_2=0} = \sum_{i=1}^{n} w_i^{IV}(y_i - \bar{y}).$$

By simple alegra and the fact that $y_i = 1$ or 0, we can get

$$\begin{aligned} U^{III} =& \sum_{i=1}^{n} w_i^{III}(y_i - \bar{y}) \\ =& \sum_{i=1}^{n} w_i^{III} y_i - \bar{y}\sum_{i=1}^{n} w_i^{III} \\ =& n_1\bar{w}_1^{III} - \frac{n_1}{n}\left(n_1\bar{w}_1^{III} + n_0\bar{w}_0^{III}\right) \\ =& \frac{n_1 n_0}{n}\left(\bar{w}_1^{III} - \bar{w}_0^{III}\right). \end{aligned}$$

Similarly, we have

$$\begin{aligned} U^{IV} =& \sum_{i=1}^{n} w_i^{IV}(y_i - \bar{y}) \\ =& \frac{n_1 n_0}{n}\left(\bar{w}_1^{IV} - \bar{w}_0^{IV}\right) \\ =& \frac{n_1 n_0}{n}\left(\bar{w}_1^{IV} - \bar{w}_0^{IV}\right). \end{aligned}$$

The ST6 test statistic $T^{VI} = \mathbf{U}_{joint}^T \hat{\Sigma}_{joint}^{-1} \mathbf{U}_{joint}$ is the quadratic form of the two score statistics U^{III} and U^{IV} for the above logistic regression, where $\mathbf{U}_{joint} = \left(U^{III}, U^{IV}\right)^T$ and $\hat{\Sigma}_{joint}$ is the estimate the covariance matrix $Cov(\mathbf{U}_{joint})$.

Note that in logistic regression (A1), y_i are random variables, while w_i^{III} and w_i^{IV} are

conditionally fixed (i.e., conditionally non-random). We can get

$$
\begin{aligned}
E\left(U^{III}\right) &= \sum_{i=1}^{n} w_i^{III} E(y_i - \bar{y}) = 0, \\
E\left(U^{IV}\right) &= \sum_{i=1}^{n} w_i^{IV} E(y_i - \bar{y}) = 0.
\end{aligned}
$$

The above equalities are true, no matter whether the null hypothesis H_0^{VI} holds or not. Hence, we have

$$
\begin{aligned}
Cov(\mathbf{U}_{joint}) &= E\left(\mathbf{U}_{joint}\mathbf{U}_{joint}^T\right) - [E(\mathbf{U}_{joint})][E(\mathbf{U}_{joint})]^T \\
&= E\left(\mathbf{U}_{joint}\mathbf{U}_{joint}^T\right) \\
&= \begin{pmatrix} E\left[\left(U^{III}\right)^2\right] & E\left[U^{III}U^{IV}\right] \\ E\left[U^{III}U^{IV}\right] & E\left[\left(U^{IV}\right)^2\right] \end{pmatrix}.
\end{aligned}
\tag{A2}
$$

We can get

$$
\begin{aligned}
\left(U^{III}\right)^2 &= \left[\sum_{i=1}^{n} w_i^{III}\left(y_i - \bar{y}\right)\right]^2 \\
&= \left[\sum_{i=1}^{n} w_i^{III} y_i - \bar{y}\sum_{i=1}^{n} w_i^{III}\right]^2 \\
&= \left(\sum_{i=1}^{n} w_i^{III} y_i\right)^2 + \bar{y}^2\left(\sum_{i=1}^{n} w_i^{III}\right)^2 - 2\left(\sum_{i=1}^{n} w_i^{III} y_i\right)\left(\bar{y}\sum_{j=1}^{n} w_j^{III}\right) \\
&= \sum_{i=1}^{n} w_i^{III} y_i \sum_{j=1}^{n} w_j^{III} y_j + \bar{y}^2\left(\sum_{i=1}^{n} w_i^{III}\right)^2 - 2\left(\sum_{j=1}^{n} w_j^{III}\right)\left(\sum_{i=1}^{n} w_i^{III} y_i \bar{y}\right) \\
&= \sum_{i=1}^{n}\sum_{j=1}^{n} w_i^{III} w_j^{III} y_i y_j + \bar{y}^2\left(\sum_{i=1}^{n} w_i^{III}\right)^2 - 2\left(\sum_{j=1}^{n} w_j^{III}\right)\left(\sum_{i=1}^{n} w_i^{III} y_i \bar{y}\right)
\end{aligned}
$$

Note that $y_i^2 = y_i$. We have

$$\begin{aligned}
&\mathrm{E}\left(\sum_{i=1}^{n}\sum_{j=1}^{n} w_i^{III} w_j^{III} y_i y_j\right) \\
=&\sum_{i=1}^{n}\sum_{j=1}^{n} w_i^{III} w_j^{III} \mathrm{E}\left(y_i y_j\right) \\
=&\sum_{i=1}^{n}\left(w_i^{III}\right)^2 \mathrm{E}\left(y_i^2\right) + \sum_{i\neq j} w_i^{III} w_j^{III} \mathrm{E}\left(y_i\right)\mathrm{E}\left(y_j\right) \\
=&\sum_{i=1}^{n}\left(w_i^{III}\right)^2 \mathrm{E}\left(y_i\right) + \sum_{i\neq j} w_i^{III} w_j^{III} \pi_i \pi_j \\
=&\sum_{i=1}^{n}\left(w_i^{III}\right)^2 \pi_i + \sum_{i\neq j} w_i^{III} w_j^{III} \pi_i \pi_j \\
=&\sum_{i=1}^{n}\left(w_i^{III}\right)^2 \pi_i - \sum_{i=1}^{n}\left(w_i^{III}\right)^2 \pi_i^2 + \sum_{i=1}^{n}\left(w_i^{III}\right)^2 \pi_i^2 + \sum_{i\neq j} w_i^{III} w_j^{III} \pi_i \pi_j \\
=&\sum_{i=1}^{n}\left(w_i^{III}\right)^2 \pi_i\left(1-\pi_i\right) \sum_{i=1}^{n}\sum_{j=1}^{n} w_i^{III} w_j^{III} \pi_i \pi_j \\
=&\sum_{i=1}^{n}\left(w_i^{III}\right)^2 \pi_i\left(1-\pi_i\right) + \left(\sum_{i=1}^{n} w_i^{III} \pi_i\right)^2 .
\end{aligned}$$

We also have

$$\begin{aligned}
\mathrm{E}\left(\bar{y}\right)^2 =&\mathrm{Var}\left(\bar{y}\right) + \left[\mathrm{E}\left(\bar{y}\right)\right]^2 \\
=&\frac{1}{n^2}\sum_{i=1}^{n}\mathrm{Var}\left(y_i\right) + \left[\frac{1}{n}\sum_{i=1}^{n}\pi_i\right]^2 \\
=&\frac{1}{n^2}\sum_{i=1}^{n}\pi_i\left(1-\pi_i\right) + \left[\frac{1}{n}\sum_{i=1}^{n}\pi_i\right]^2
\end{aligned} \tag{A3}$$

And

$$
\begin{aligned}
\mathrm{E}\left(\sum_{i=1}^{n} w_i^{III} y_i \bar{y}\right) =& \sum_{i=1}^{n} w_i^{III} \mathrm{E}\left(y_i \bar{y}\right) \\
=& \sum_{i=1}^{n} w_i^{III} \mathrm{E}\left[y_i \frac{1}{n} \sum_{j=1}^{n} y_j\right] \\
=& \frac{1}{n} \sum_{i=1}^{n} w_i^{III} \sum_{j=1}^{n} \mathrm{E}\left(y_i y_j\right) \\
=& \frac{1}{n} \sum_{i=1}^{n} w_i^{III} \left[\sum_{j=1}^{n} \mathrm{E}\left(y_i y_j\right)\right] \\
=& \frac{1}{n} \sum_{i=1}^{n} w_i^{III} \left[\mathrm{E}\left(y_i^2\right) + \sum_{j=1, j\neq i}^{n} \mathrm{E}\left(y_i\right) \mathrm{E}\left(y_j\right)\right] \\
=& \frac{1}{n} \sum_{i=1}^{n} w_i^{III} \left[\mathrm{E}\left(y_i\right) + \sum_{j=1, j\neq i}^{n} \pi_i \pi_j\right] \\
=& \frac{1}{n} \sum_{i=1}^{n} w_i^{III} \left[\pi_i + \sum_{j=1, j\neq i}^{n} \pi_i \pi_j\right] \\
=& \frac{1}{n} \sum_{i=1}^{n} w_i^{III} \left[\pi_i - \pi_i^2 + \pi_i^2 + \sum_{j=1, j\neq i}^{n} \pi_i \pi_j\right] \\
=& \frac{1}{n} \sum_{i=1}^{n} w_i^{III} \left[\pi_i \left(1 - \pi_i\right) + \sum_{j=1}^{n} \pi_i \pi_j\right] \\
=& \frac{1}{n} \sum_{i=1}^{n} w_i^{III} \left[\pi_i \left(1 - \pi_i\right) + \pi_i \sum_{j=1}^{n} \pi_j\right]
\end{aligned}
$$

Hence,

$$
\begin{aligned}
\mathrm{E}\left[\left(U^{III}\right)^2\right] =& \sum_{i=1}^{n} \sum_{j=1}^{n} w_i^{III} w_j^{III} \mathrm{E}\left(y_i y_j\right) + \mathrm{E}\left(\bar{y}^2\right) \left(\sum_{i=1}^{n} w_i^{III}\right)^2 - 2\left(\sum_{j=1}^{n} w_j^{III}\right) \sum_{i=1}^{n} w_i^{III} \mathrm{E}\left(y_i \bar{y}\right) \\
=& \sum_{i=1}^{n} \left(w_i^{III}\right)^2 \pi_i \left(1 - \pi_i\right) + \left(\sum_{i=1}^{n} w_i^{III} \pi_i\right)^2 \\
&+ \left[\frac{1}{n^2} \sum_{i=1}^{n} \pi_i \left(1 - \pi_i\right) + \left(\frac{1}{n} \sum_{i=1}^{n} \pi_i\right)^2\right] \left(\sum_{i=1}^{n} w_i^{III}\right)^2 \\
&- 2\left(\sum_{j=1}^{n} w_j^{III}\right) \frac{1}{n} \sum_{i=1}^{n} w_i^{III} \left[\pi_i \left(1 - \pi_i\right) + \pi_i \sum_{j=1}^{n} \pi_j\right]
\end{aligned}
\tag{A4}
$$

Under $H_0^{VI}: \gamma_1 = \gamma_2 = 0$, we can estimate π_0 by $\bar{y} = n_1/n$ and can estimate $\mathrm{E}\left(U^{III}\right)^2$

by

$$
\begin{aligned}
\widehat{\mathrm{E}}\left(U^{III}|H_0^{VI}\right)^2 =& \sum_{i=1}^{n}\left(w_i^{III}\right)^2 \frac{n_1}{n}\left(1-\frac{n_1}{n}\right)+\left(\sum_{i=1}^{n} w_i^{III}\frac{n_1}{n}\right)^2 \\
&+\left[\frac{1}{n^2}\sum_{i=1}^{n}\frac{n_1}{n}\left(1-\frac{n_1}{n}\right)+\left(\frac{1}{n}\sum_{i=1}^{n}\frac{n_1}{n}\right)^2\right]\left(\sum_{i=1}^{n} w_i^{III}\right)^2 \\
&-2\left(\sum_{j=1}^{n} w_j^{III}\right)\frac{1}{n}\sum_{i=1}^{n} w_i^{III}\left[\frac{n_1}{n}\left(1-\frac{n_1}{n}\right)+\frac{n_1}{n}\sum_{j=1}^{n}\frac{n_1}{n}\right] \\
=& \frac{n_1}{n}\frac{n_0}{n}\sum_{i=1}^{n}\left(w_i^{III}\right)^2+\frac{n_1^2}{n^2}\left(\sum_{i=1}^{n} w_i^{III}\right)^2 \\
&+\left[\frac{1}{n^2}\frac{n_1}{n}\frac{n_0}{n}n+\frac{1}{n^2}\frac{n_1^2}{n^2}n^2\right]\left(\sum_{i=1}^{n} w_i^{III}\right)^2 \\
&-\frac{2}{n}\left(\sum_{j=1}^{n} w_j^{III}\right)^2\left[\frac{n_1 n_0}{n^2}+\frac{n_1^2}{n^2}n\right] \\
=& \frac{n_1}{n}\frac{n_0}{n}\sum_{i=1}^{n}\left(w_i^{III}\right)^2+\left(\sum_{i=1}^{n} w_i^{III}\right)^2\left[\frac{n_1^2}{n^2}+\frac{n_1 n_0}{n^3}+\frac{n_1^2}{n^2}-2\frac{n_1 n_0}{n^3}-2\frac{n_1^2}{n^2}\right] \\
=& \frac{n_1}{n}\frac{n_0}{n}\sum_{i=1}^{n}\left(w_i^{III}\right)^2-\frac{n_1 n_0}{n^3}\left(\sum_{i=1}^{n} w_i^{III}\right)^2 \\
=& \frac{n_1}{n}\frac{n_0}{n}\left[\sum_{i=1}^{n}\left(w_i^{III}\right)^2-\frac{1}{n}\left(\sum_{i=1}^{n} w_i^{III}\right)^2\right] \\
=& \bar{y}\left(1-\bar{y}\right)\sum_{i=1}^{n}\left(w_i^{III}-\bar{w}^{III}\right)^2 .
\end{aligned}
$$

That is,

$$
V\hat{a}r\left(U^{III}|H_0^{VI}\right)=\hat{E}\left[\left(U^{III}\right)^2|H_0^{VI}\right]=\bar{y}\left(1-\bar{y}\right)\sum_{i=1}^{n}\left(w_i^{III}-\bar{w}^{III}\right)^2 .
$$

Similarly, we can estimate $Var\left(U^{IV}\right)$ by

$$
V\hat{a}r\left(U^{IV}|H_0^{VI}\right)=\hat{E}\left[\left(U^{IV}\right)^2|H_0^{VI}\right]=\bar{y}\left(1-\bar{y}\right)\sum_{i=1}^{n}\left(w_i^{IV}-\bar{w}^{IV}\right)^2 .
$$

Next, we calculate $E\left(U^{III}U^{IV}\right)$.

$$
\begin{aligned}
\mathrm{E}\left[U^{III}U^{IV}\right] =& \mathrm{E}\left[\sum_{i=1}^{n} w_i^{III}\left(y_i-\bar{y}\right)\sum_{j=1}^{n} w_j^{IV}\left(y_j-\bar{y}\right)\right] \\
=& \sum_{i=1}^{n}\sum_{j=1}^{n} w_i^{III} w^{IV} \mathrm{E}\left[\left(y_i-\bar{y}\right)\left(y_j-\bar{y}\right)\right] \\
=& \sum_{i=j} w_i^{III} w_i^{IV} \mathrm{E}\left[\left(y_i-\bar{y}\right)^2\right] \\
& + \sum_{i\neq j} w_i^{III} w^{IV} \mathrm{E}\left[\left(y_i-\bar{y}\right)\left(y_j-\bar{y}\right)\right]
\end{aligned}
$$

Note that $y_i^2 = y_i$ since y_i is binary variable taking values 1 or 0. We can calculate

$$
\begin{aligned}
\mathrm{E}\left[\left(y_i-\bar{y}\right)^2\right] =& \mathrm{E}\left[y_i^2+\bar{y}^2-2y_i\bar{y}\right] \\
=& \mathrm{E}\left[y_i+\bar{y}^2-2y_i\bar{y}\right] \\
=& \mathrm{E}\left(y_i\right)+\mathrm{E}\left(\bar{y}^2\right)-2\mathrm{E}\left(y_i\bar{y}\right)
\end{aligned}
$$

We have $\mathrm{E}\,(y_i) = \pi_i$. Based on Formula (A3), we also can calculate

$$\begin{aligned}
\mathrm{E}\,(y_i\bar{y}) =& \mathrm{E}\left(y_i \frac{1}{n}\sum_{k=1}^{n} y_k\right)\\
=& \frac{1}{n}\sum_{k=1}^{n}\mathrm{E}\,(y_i y_k)\\
=& \frac{1}{n}\left[\mathrm{E}\left(y_i^2\right) + \sum_{k=1,k\neq i}^{n}\mathrm{E}\,(y_i y_k)\right]\\
=& \frac{1}{n}\left[\mathrm{E}\,(y_i) + \sum_{k=1,k\neq i}^{n}\mathrm{E}\,(y_i)\,\mathrm{E}\,(y_k)\right]\\
=& \frac{1}{n}\left[\pi_i + \sum_{k=1,k\neq i}^{n}\pi_i\pi_k\right]\\
=& \frac{1}{n}\left[\pi_i + \pi_i\sum_{k=1,k\neq i}^{n}\pi_k\right]\\
=& \frac{1}{n}\left\{\pi_i + \pi_i\left[\sum_{k=1}^{n}\pi_k - \pi_i\right]\right\}\\
=& \frac{1}{n}\left\{\pi_i\left(1-\pi_i\right) + \pi_i\sum_{k=1}^{n}\pi_k\right\}
\end{aligned}$$

Hence, we can get

$$\begin{aligned}
\mathrm{E}\,(y_i - \bar{y})^2 =& \pi_i + \frac{1}{n^2}\sum_{k=1}^{n}\pi_k\left(1-\pi_k\right) + \left[\frac{1}{n}\sum_{k=1}^{n}\pi_k\right]^2\\
&- \frac{2}{n}\left[\pi_i\left(1-\pi_i\right) + \pi_i\sum_{k=1}^{n}\pi_k\right]
\end{aligned} \tag{A5}$$

We next calculate $\mathrm{E}\left(y_i-\bar{y}\right)\left(y_j-\bar{y}\right)$ for $i \neq j$:

$$\begin{aligned}
&\mathrm{E}\left(y_i-\bar{y}\right)\left(y_j-\bar{y}\right) \\
=&\mathrm{E}\left(y_i y_j-y_i \bar{y}-y_j \bar{y}+\bar{y}^2\right) \\
=&\mathrm{E}\left(y_i\right) \mathrm{E}\left(y_j\right)-\mathrm{E}\left(y_i \bar{y}\right)-\mathrm{E}\left(y_j \bar{y}\right)+\mathrm{E}\left(\bar{y}^2\right) \\
=&\pi_i \pi_j-\frac{1}{n}\left[\pi_i\left(1-\pi_i\right)+\pi_i \sum_{k=1}^n \pi_k\right]-\frac{1}{n}\left[\pi_j\left(1-\pi_j\right)+\pi_j \sum_{k=1}^n \pi_k\right] \\
&+\frac{1}{n^2} \sum_{k=1}^n \pi_k\left(1-\pi_k\right)+\left[\frac{1}{n} \sum_{k=1}^n \pi_k\right]^2
\end{aligned} \tag{A6}$$

Therefore, we can get

$$\begin{aligned}
\mathrm{E}\left(U^{III} U^{IV}\right)=&\sum_{i=1}^n w_i^{III} w_i^{IV}\left\{\pi_i+\frac{1}{n^2} \sum_{k=1}^n \pi_k\left(1-\pi_k\right)+\left[\frac{1}{n} \sum_{k=1}^n \pi_k\right]^2\right. \\
&\left.-\frac{2}{n}\left[\pi_i\left(1-\pi_i\right)+\pi_i \sum_{k=1}^n \pi_k\right]\right\} \\
&+\sum_{i \neq j} w_i^{III} w^{IV}\left\{\pi_i \pi_j-\frac{1}{n}\left[\pi_i\left(1-\pi_i\right)+\pi_i \sum_{k=1}^n \pi_k\right]\right. \\
&-\frac{1}{n}\left[\pi_j\left(1-\pi_j\right)+\pi_j \sum_{k=1}^n \pi_k\right] \\
&\left.+\frac{1}{n^2} \sum_{k=1}^n \pi_k\left(1-\pi_k\right)+\left[\frac{1}{n} \sum_{k=1}^n \pi_k\right]^2\right\}
\end{aligned} \tag{A7}$$

Therefore we then can get under H_0^{VI}

$$
\begin{aligned}
\widehat{\mathrm{E}}\left[U^{III}U^{IV}\right] \overset{H_0^{VI}}{=} & \sum_{i=1}^{n} w_i^{III} w_i^{IV} \Bigg\{ \bar{y} + \frac{1}{n^2}\sum_{k=1}^{n} \bar{y}\left(1-\bar{y}\right) + \left[\frac{1}{n}\sum_{k=1}^{n}\bar{y}\right]^2 \\
& -\frac{2}{n}\left[\bar{y}\left(1-\bar{y}\right) + \bar{y}\sum_{k=1}^{n}\bar{y}\right]\Bigg\} \\
& + \sum_{i\neq j} w_i^{III} w_j^{IV} \Bigg\{ \bar{y}\bar{y} - \frac{1}{n}\left[\bar{y}\left(1-\bar{y}\right) + \bar{y}\sum_{k=1}^{n}\bar{y}\right] - \frac{1}{n}\left[\bar{y}\left(1-\bar{y}\right) + \bar{y}\sum_{k=1}^{n}\bar{y}\right] \\
& + \frac{1}{n^2}\sum_{k=1}^{n}\bar{y}\left(1-\bar{y}\right) + \left[\frac{1}{n}\sum_{k=1}^{n}\bar{y}\right]^2 \Bigg\} \\
= & \sum_{i=1}^{n} w_i^{III} w_i^{IV} \left\{ \bar{y} + \frac{1}{n}\bar{y}\left(1-\bar{y}\right) + \bar{y}^2 - \frac{2}{n}\bar{y}\left(1-\bar{y}\right) - 2\bar{y}^2 \right\} \\
& + \sum_{i\neq j} w_i^{III} w_j^{IV} \left\{ \bar{y}^2 - \frac{1}{n}\bar{y}\left(1-\bar{y}\right) - \bar{y}^2 - \frac{1}{n}\bar{y}\left(1-\bar{y}\right) - \bar{y}^2 + \frac{1}{n}\bar{y}\left(1-\bar{y}\right) + \bar{y}^2 \right\} \\
= & \sum_{i=1}^{n} w_i^{III} w_i^{IV} \left\{ \bar{y} - \bar{y}^2 - \frac{1}{n}\bar{y}\left(1-\bar{y}\right) \right\} - \frac{1}{n}\bar{y}\left(1-\bar{y}\right)\sum_{i\neq j} w_i^{III} w_j^{IV} \\
= & \bar{y}\left(1-\bar{y}\right)\sum_{i=1}^{n} w_i^{III} w_i^{IV} - \frac{1}{n}\bar{y}\left(1-\bar{y}\right)\left[\sum_{i=1}^{n} w_i^{III} w_i^{IV} + \sum_{i\neq j} w_i^{III} w_j^{IV}\right] \\
= & \bar{y}\left(1-\bar{y}\right)\sum_{i=1}^{n} w_i^{III} w_i^{IV} - \frac{1}{n}\bar{y}\left(1-\bar{y}\right)\sum_{i=1}^{n} w_i^{III}\sum_{j=1}^{n} w_j^{IV} \\
= & \bar{y}\left(1-\bar{y}\right)\left[\sum_{i=1}^{n} w_i^{III} w_i^{IV} - \frac{1}{n}\sum_{i=1}^{n} w_i^{III}\sum_{j=1}^{n} w_j^{IV}\right] \\
= & \bar{y}\left(1-\bar{y}\right)\left[\sum_{i=1}^{n} w_i^{III} w_i^{IV} - n\bar{w}^{III}\bar{w}^{IV}\right] \\
= & \bar{y}\left(1-\bar{y}\right)\left[\sum_{i=1}^{n} \left(w_i^{III} - \bar{w}^{III}\right)\left(w_i^{IV} - \bar{w}^{IV}\right)\right]
\end{aligned}
$$

Therefore, we have

$$\widehat{\text{Cov}}\left(\mathbf{U}_{joint}\right) \overset{H_0^{VI}}{=} \bar{y}\left(1-\bar{y}\right)\begin{pmatrix} \sum_{i=1}^{n}\left(w_i^{III}-\bar{w}^{III}\right)^2 & \sum_{i=1}^{n}\left(w_i^{III}-\bar{w}^{III}\right)\left(w_i^{IV}-\bar{w}^{IV}\right) \\ \sum_{i=1}^{n}\left(w_i^{III}-\bar{w}^{III}\right)\left(w_i^{IV}-\bar{w}^{IV}\right) & \sum_{j=1}^{n}\left(w^{IV}-\bar{w}^{IV}\right)^2 \end{pmatrix}$$

$$= n\bar{y}\left(1-\bar{y}\right)\begin{pmatrix} \hat{\sigma}^2_{w^{III}} & \hat{\sigma}_{w^{III}w^{IV}} \\ \hat{\sigma}_{w^{III}w^{IV}} & \hat{\sigma}^2_{w^{IV}} \end{pmatrix},$$

where $\hat{\sigma}^2_{w^{III}} = \sum_{i=1}^{n}(w_i^{III}-\bar{w}^{III})^2/n$ and $\hat{\sigma}^2_{w^{IV}} = \sum_{i=1}^{n}(w_i^{IV}-\bar{w}^{IV})^2/n$ are the sample variances for w_i^{III} and w_i^{IV}, and $\hat{\sigma}_{w^{III}w^{IV}} = \sum_{i=1}^{n}(w_i^{III}-\bar{w}^{III})(w_i^{IV}-\bar{w}^{IV})/n$ is the sample covariance between w_i^{III} and w_i^{IV}.

Note that in logistic regression (A1), the random variables are y_i, while w_i^{III} and w_i^{IV} are conditionally fixed (i.e., conditionally non-random). Hence, the (asymptotic) distributions of the U^{III}, U^{IV}, and T_{joint} do not depend on the distributions of w_i^{III} and w_i^{IV}. In this sense, we can say that the joint statistic T_{joint} are robust to the violation of the normality assumptions for the predictors w_i^{III} and w_i^{IV}.

Based on Dobson (1990),

$$\mathbf{U}_{joint} \overset{H_0^{VI}}{\to} N(0, Cov(\mathbf{U}_{joint})).$$

Denote $\mathbf{\Omega} = Cov(\mathbf{U}_{joint}|H_0^{VI})$. We have

$$\mathbf{\Omega}^{-1/2}\mathbf{U}_{joint} \overset{H_0^{VI}}{\to} N(0, \mathbf{I}_2).$$

By the relationship beween multivariate normal distribution and chi square distribution, we have

$$\left(\mathbf{\Omega}^{-1/2}\mathbf{U}_{joint}\right)^T\left(\mathbf{\Omega}^{-1/2}\mathbf{U}_{joint}\right) = \mathbf{U}_{joint}^T\mathbf{\Omega}^{-1}\mathbf{U}_{joint} \overset{H_0^{VI}}{\to} \chi^2_2.$$

Based on the Law of Large Numbers, we have

$$\widehat{Cov}(\mathbf{U}_{joint}) \stackrel{H_0^{VI}}{\rightarrow} Cov(\mathbf{U}_{joint}).$$

Hence, we have

$$T_{joint} = \mathbf{U}_{joint}^T \left[\widehat{Cov}\left(\mathbf{U}_{joint}\right)\right]^{-1} \mathbf{U}_{joint} \stackrel{H_0^{VI}}{\rightarrow} \chi_2^2. \tag{A8}$$

Note that we can derive an estimate of $Cov(\mathbf{U}_{joint})$ under the alternative hypothesis based on formulas (A2), (A4), and (A7).

B The asymptotic distribution of the ST6 test statistic when $\widehat{Cov}\left(\mathbf{U}_{joint}\right)$ is not full rank

When the rank of $\widehat{Cov}\left(\mathbf{U}_{joint}\right)$ is one, which lead to non existence of $\left[\widehat{Cov}\left(\mathbf{U}_{joint}\right)\right]^{-1}$, we replace the inverse of $\widehat{Cov}\left(\mathbf{U}_{joint}\right)$ by its Penrose-Moore generalized inverse and we have

$$T_{joint} = \mathbf{U}_{joint}^T \left[\widehat{Cov}\left(\mathbf{U}_{joint}\right)\right]^{+} \mathbf{U}_{joint} \stackrel{H_0^{VI}}{\rightarrow} \chi_1^2, \tag{A9}$$

where $\left[\widehat{Cov}\left(\mathbf{U}_{joint}\right)\right]^{+}$ is the Penrose-Moore generalized inverse of $\widehat{Cov}\left(\mathbf{U}_{joint}\right)$.

References

Dobson, Annette J. (1990). *An Introduction to Generalized Linear Models*. Chapman and Hall.
